# Quantifying variations associated with dental caries reveals disparity in effect allele frequencies across diverse populations

**DOI:** 10.1186/s12863-024-01215-z

**Published:** 2024-06-03

**Authors:** Sangram Sandhu, Varun Sharma, Sachin Kumar, Niraj Rai, Pooran Chand

**Affiliations:** 1 Ancient DNA Lab, Birbal Sahni Institute of Palaeosciences, 226607 Lucknow, Uttar Pradesh India; 2 Department of Prosthodontics, Faculty of Dental Sciences, King George’s Medical University, 226003 Lucknow, India; 3 NMC Genetics India Pvt Ltd, 122002 Gurugram, Haryana India; 4 Academy of Scientific and Innovative Research (AcSIR), 201002 Ghaziabad, India

**Keywords:** Dental Caries (DC), GWAS, Alleles frequencies, FST, Genetic affinity, Ethnicities

## Abstract

**Background:**

Dental caries (DC) is a multifaceted oral condition influenced by genetic and environmental factors. Recent advancements in genotyping and sequencing technologies, such as Genome-Wide Association Studies (GWAS) have helped researchers to identify numerous genetic variants associated with DC, but their prevalence and significance across diverse global populations remain poorly understood as most of the studies were conducted in European populations, and very few were conducted in Asians specifically in Indians.

**Aim:**

This study aimed to evaluate the genetic affinity of effect alleles associated with DC to understand the genetic relationship between global populations with respect to the Indian context.

**Methodology:**

This present study used an empirical approach in which variants associated with DC susceptibility were selected. These variants were identified and annotated using the GWAS summary. The genetic affinity was evaluated using *Fst*.

**Results:**

The effect of allele frequencies among different populations was examined, revealing variations in allele distribution. African populations exhibited higher frequencies of specific risk alleles, whereas East Asian and European populations displayed distinct profiles. South Asian populations showed a unique genetic cluster.

**Conclusion:**

Our study emphasises the complex genetic landscape of DC and highlights the need for population-specific research as well as validation of GWAS-identified markers in Indians before defining them as established candidate genes.

**Supplementary Information:**

The online version contains supplementary material available at 10.1186/s12863-024-01215-z.

## Introduction

DC is a multifactorial and dynamic condition that causes demineralisation of dental hard tissues [1]. DC is triggered by acid-producing bacteria such as *Streptococcus mutans* [2] and *Lactobacillus* [3–5]. This production of acid results in the development of tiny cavities, which can progress deeper, affecting the dentin and pulp if left untreated [6, 7]. A diet high in carbohydrates provides the necessary substrate for cariogenic bacteria to produce acid [8]. Moreover, inadequate oral hygiene practices, such as irregular brushing and flossing, trigger bacterial growth and acid production [9]. Several factors contribute to the development of dental caries: microbiota [9], diet [10], oral hygiene [11] and genetics [12]. This genetic predisposition is related to gene variations associated with enamel formation, saliva composition, immune response and host gene interaction mechanisms. Among these factors, the genetics of DC have been less studied.

Over the years, researchers have conducted Genome-Wide Association Studies (GWAS) to identify variations associated with dental caries susceptibility [13–15]. These studies have shed light on the genetic components contributing to an individual’s risk of developing DC [16]. Interestingly, many variants discovered through GWAS were novel, with a limited number successfully replicated in other population groups. This observation underscores the complicated nature of the DC progression. Variations within these genes have been linked to enamel mineralisation and depletion, which can impact an individual’s susceptibility to dental caries. Variations in genes related to immune response [17, 18] have been linked to variations in the host’s ability to control bacterial growth in the oral cavity. Some GWAS studies have explored the genetic variations in *Streptococcus mutans* itself [14], the primary cariogenic bacteria. SNPs in *Streptococcus mutans* adherence genes may affect the bacterium’s ability to colonise and adhere to tooth surfaces [19].

Billions of individuals are impacted worldwide by DC, as revealed by the global burden of disease [20]. DC poses a substantial global challenge if left unaddressed, affecting over 34 million people [21]. However, with the advent of array-based genome-wide studies (GWAS) and sequencing technologies, evidence supporting the role of genetics in DC is steadily expanding [13, 14, 22–28]. Interestingly, many variants discovered through GWAS were novel, with a limited number successfully replicated in other genetic studies. The variants should be replicated or validated in other ethnicities as because most studies have focused on European and other populations but absent in South Asians, especially in Indians. This observation underscores the complicated nature of the genetics behind dental caries. Furthermore, the disparities in effect allele frequencies across diverse populations indicate that the genetic factors contributing to dental caries can vary significantly between different ethnic and geographic groups, highlighting the need for tailored approaches to prevention and treatment. With this observation, we aim to evaluate the genetic affinity of effect alleles associated with DC among globally diverse populations which may provide valuable insights into the genetic aspects of DC and its potential underlying causes.

## Methodology

**Fig. 1:**
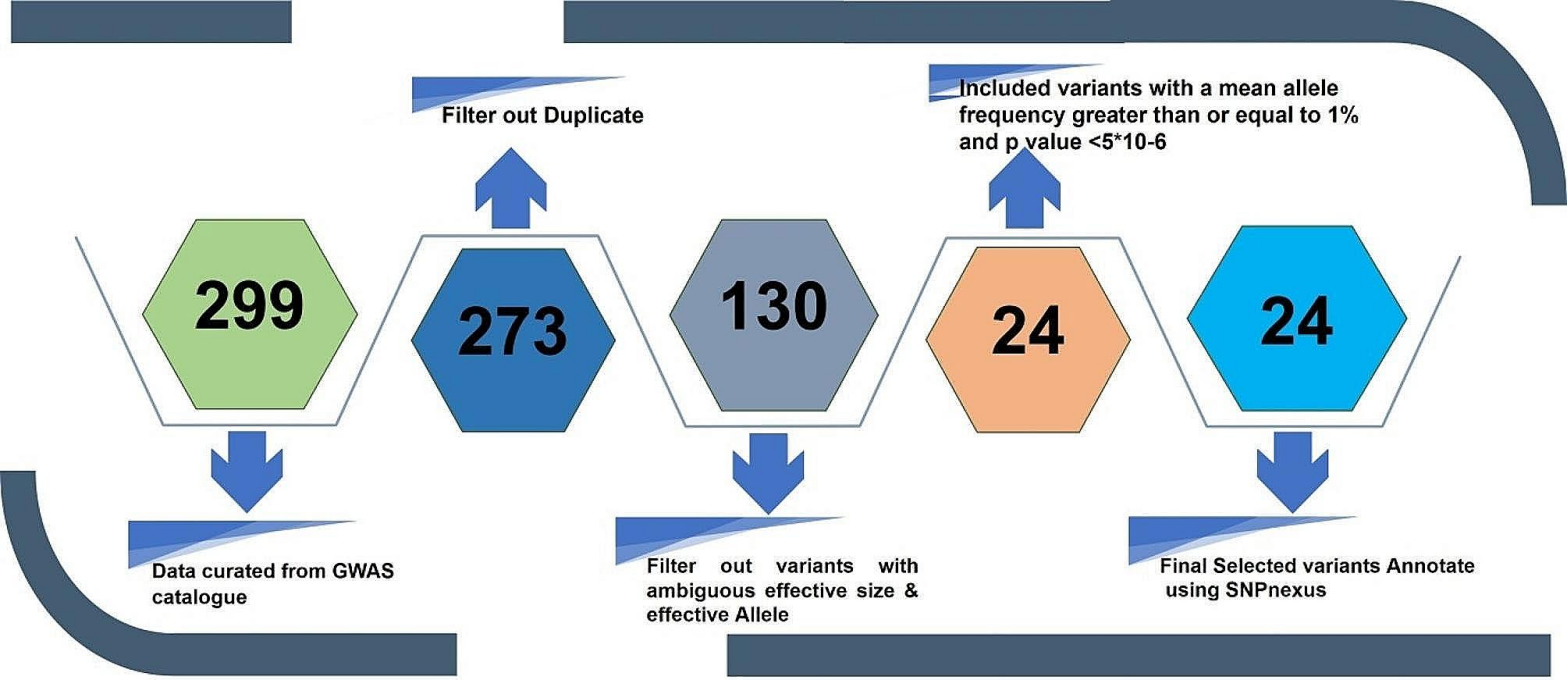
The flowchart depiction of the methodology employed for the screening of variants from the GWAS catalogue

The present study is an empirical approach to understanding the genetic difference processed by the variations associated with DC. The variants were curated from the GWAS catalogue (www.ebi.ac.uk/gwas). The selected variations have also shown their association with traits associated with DC, such as Pit-and-Fissure caries, paediatric dental caries, early childhood caries, primary dental caries, smooth-surface caries, etc. The criteria for screening and selecting the variants associated with DC have been summarised in Fig. 1. Our initial curation yielded 273 unique variants (Supplementary Table 1).

**Fig. 2:**
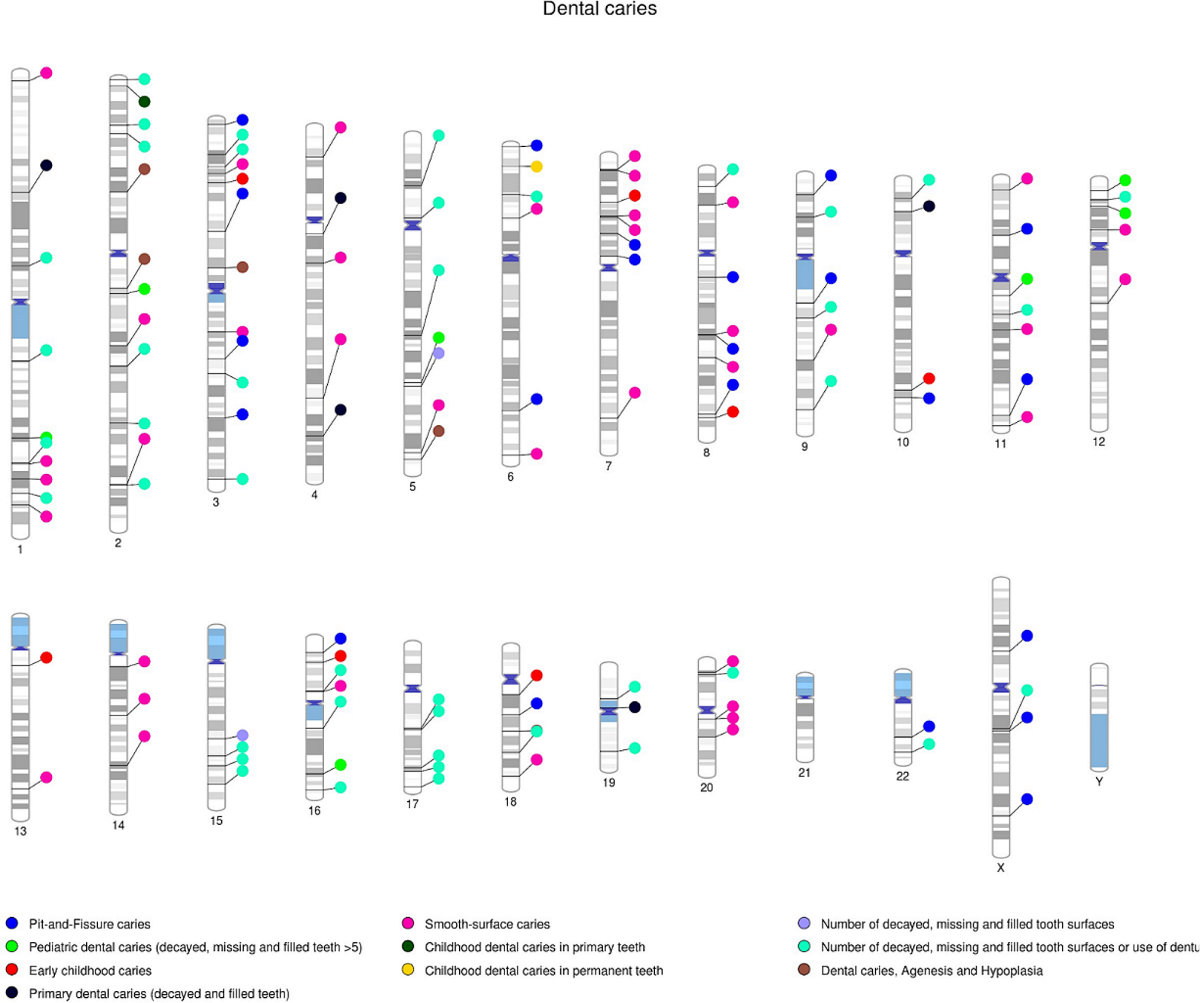
Phenogram illustrating the genomic distribution of 130 variants across different human chromosomes. These subtypes reflect closely related traits, collectively constituting the diverse spectrum of genetic factors influencing dental caries risk in its various clinical presentations. The phenotype

To further refine our selection, we included variants with a mean effect allele frequency of ≥ 1% in Europeans (EUR), Admixed Americans (AA), Africans (AFR), East Asians (EAS) and South Asians (SAS).Variations with a mean MAF more significant than 1% warrant a comprehensive reporting of genetic diversity, encompassing both common and rarer variants in the overall analysis. A threshold of greater than 1% for the mean effect allele frequency in population groups helps to achive a balance between statistical power and specificity. Moreover, the variations with ambiguous markers with undefined effect alleles and no effect size values and odds were removed, and a total of 130 variants (Supplementary Table 2) were selected. A phylogram of filter-out 130 variants with their chromosomal location and possible associated traits is summarised in **(**Fig. 2**)**. The second criterion for filtering variations was to screen variations with a p-value threshold of less than 5 × 10^− 6^ and to meet other frequency and annotation criteria were to maximize the number of potentially functional polymorphisms for analysing allele frequency differences and genetic distances across diverse populations. Filtering strictly based on the GWAS threshold alone would result in a reduction of potential variations, especially considering that many variants of significance fall within the range of 10^− 6^ to 10^− 7^ (Supplementary Table 2).

This screening left 24 variants for the analysis (Table 1). From the prediction effect analysis, out of 24 variants. The selected final SNPs were annotated using SNP nexus (https://www.snp-nexus.org/v4/).

**Table 1:** Final variations included for the downstream analysis with mean allele frequencies

Variation ID	Chr	Position	dbSNP	REF Allele	ALT Allele	Minor Allele	Gene(s)	Mean Allele Freq
rs10495332	chr1	232,620,280	rs10495332	T	C	C	SIPA1L2	0.4449
rs1079204	chr2	218,265,791	rs1079204	G	A	A	AAMP	0.1291
rs121908120	chr2	218,890,289	rs121908120	T	A	A	WNT10A	0.0329
rs4016429	chr3	28,923,310	rs4016429	C	G	G	RBMS3	0.4491
rs9311745	chr3	60,016,099	rs9311745	T	C	C	FHIT	0.8257
rs17236529	chr3	160,508,519	rs17236529	C	T	T	KPNA4	0.047
rs185566659	chr3	193,676,936	rs185566659	G	A	A	OPA1	0.1132
rs6818964	chr4	73,104,747	rs6818964	C	T	T	ANKRD17	0.1252
rs9471075	chr6	39,339,256	rs9471075	A	C	C	KIF6	0.5605
rs197480	chr6	142,749,368	rs197480	C	T	T	HIVEP2,ADGRG6	0.0466
rs3798305	chr6	166,949,985	rs3798305	C	T	T	RNASET2	0.2531
rs6953213	chr7	7,273,854	rs6953213	G	A	A	COL28A1,C1GALT1	0.3604
rs215738	chr7	32,404,823	rs215738	G	A	G	PDE1C	0.41747
rs16882396	chr7	141,188,521	rs16882396	C	G	G	TMEM178B	0.2252
rs17128007	chr8	19,225,642	rs17128007	C	T	T	SH2D4A	0.1474
rs16867579	chr8	101,363,039	rs16867579	A	C	C	NACA4P	0.5031
rs10958998	chr9	10,275,798	rs10958998	A	G	G	PTPRD	0.5043
rs149467613	chr11	73,232,438	rs149467613	G	A	A	P2RY2	0.1135
rs16954776	chr13	98,048,363	rs16954776	A	G	G	IPO5,FARP1	0.5054
rs2877649	chr14	24,484,796	rs2877649	T	C	T	CMA1,SDR39U1	0.4243
rs1064524	chr16	30,481,502	rs1064524	C	T	T	ITGAL	0.0641
rs17088439	chr18	73,854,856	rs17088439	T	G	G	-	0.5764
rs6120141	chr20	33,180,849	rs6120141	T	C	C	BPIFA2	0.628
rs17124372	chr20	33,210,631	rs17124372	T	C	C	BPIFA3,BPIFA4P	0.2844

Allele population frequencies of selected variations were obtained from the 1000 Genomes database, which is accessible at www.internationalgenome.org. Our study encompassed diverse populations, each meticulously selected for comprehensive allele frequency analysis. These populations encompassed both pooled and stratified analyses, classified as Africa (AFR), South Asia (SAS), Admixed American (AMR), East Asia (EAS), and Europe (EUR). Within the South Asian (SAS) category, we further dissected subpopulations, including Sri Lankan Tamil from the U.K. (STU), Bengali from Bangladesh (BEB), Gujarati Indian from Houston (GIH), Indian Telugu from the U.K. (ITU), and Punjabi from Lahore, Pakistan (PJL). Similarly, within the East Asian (EAS) category, we meticulously considered Southern Han Chinese (CHS), Japanese in Tokyo (JPT), and Chinese Dai in Xishuangbanna, China (CDX). The European (EUR) population includes the groups of Finnish (FIN) and British in England and Scotland (GBR).

Subsequently, we conducted advanced statistical analyses anchored in these allele frequencies. FST (Fixation Index) values, crucial for understanding genetic distances among various population groups, were calculated using Arlequin version 3.5.2.2, supplemented with 10,000 permutations to mitigate the risk of spurious discoveries. Slatkin’s linearised F_ST_ model was then used to compute the average pairwise genetic differences, offering invaluable insights into population structure [29]. Fst values were used to produce multi dimensional scaling plot. ggplot2 from R language packages was used to visualise results (https://cran.r-project.org/web/packages/ggplot2/index.html.).

## Results

The data was analysed for twenty-four variations that surpassed the filtering criteria in this study. All SNPs selected in the study have a mean frequency of ≥ 1%. The selected variations were further annotated; out of twenty-four variations. The allele frequencies of the final twenty-four genetic variants were systematically examined across three comprehensive genomic databases: 1000 Genomes (1000G), gnomAD, and the Human Genome Diversity Project (HGDP). A focused investigation was conducted to elucidate the genetic affinities inherent in these datasets among distinct population groups, namely East Asians, South Asians, Europeans, Admixed Americans, and African populations.

This observation suggests a high degree of concordance in the genetic makeup represented by the examined variants across diverse global populations.

Additionally, functional annotations were applied to seventeen of these genetic variants, indicating their potential involvement in functional processes (Supplementary Table 4). This suggests that these genetic changes may contribute to an elevated risk of DC. Furthermore, an assessment of the epigenetic effects of selected genetic variations were analysed (Supplementary Table 5). Interestingly, it was observed that the histone modifications (H3K4me1, H3K36me3, H3K27me3, H4K20me1) effect of these selected variations, suggesting that these variations may play a crucial role in the epigenetic regulation of neighbouring genes and may potentially affect the cellular processes associated with DC. Moreover, the epigenetic modifications were also observed to be associated with specific cell types/tissues, including bipolar neurons, CD14 positive monocytes, K562 cells, B cells, and many more, indicating that the epigenetic effects of these variations may be context-dependent and could impact gene expression in different cell types. The variants may have implications for immune system function, whereas those associated with bipolar neurons may be relevant to neurological processes. This complex functional variability of selected variation and its association with DC underscores that their effect may vary in different population groups. To understand the differences in function variability, estimating the genetic affinity different population groups hold becomes pertinent.

The risk allele frequencies were compared with respect to super population and subpopulation Supplementary Figure S1). Out of twenty-four filter variants 7 has the highest frequencies in African population, and five and three have high frequencies among Europeans and East Asian populations. In terms of the South Asian population only three variants have high frequencies, but they are less detrimental as compared to other populations (Supplementary Figure S1).

**Fig. 3:**
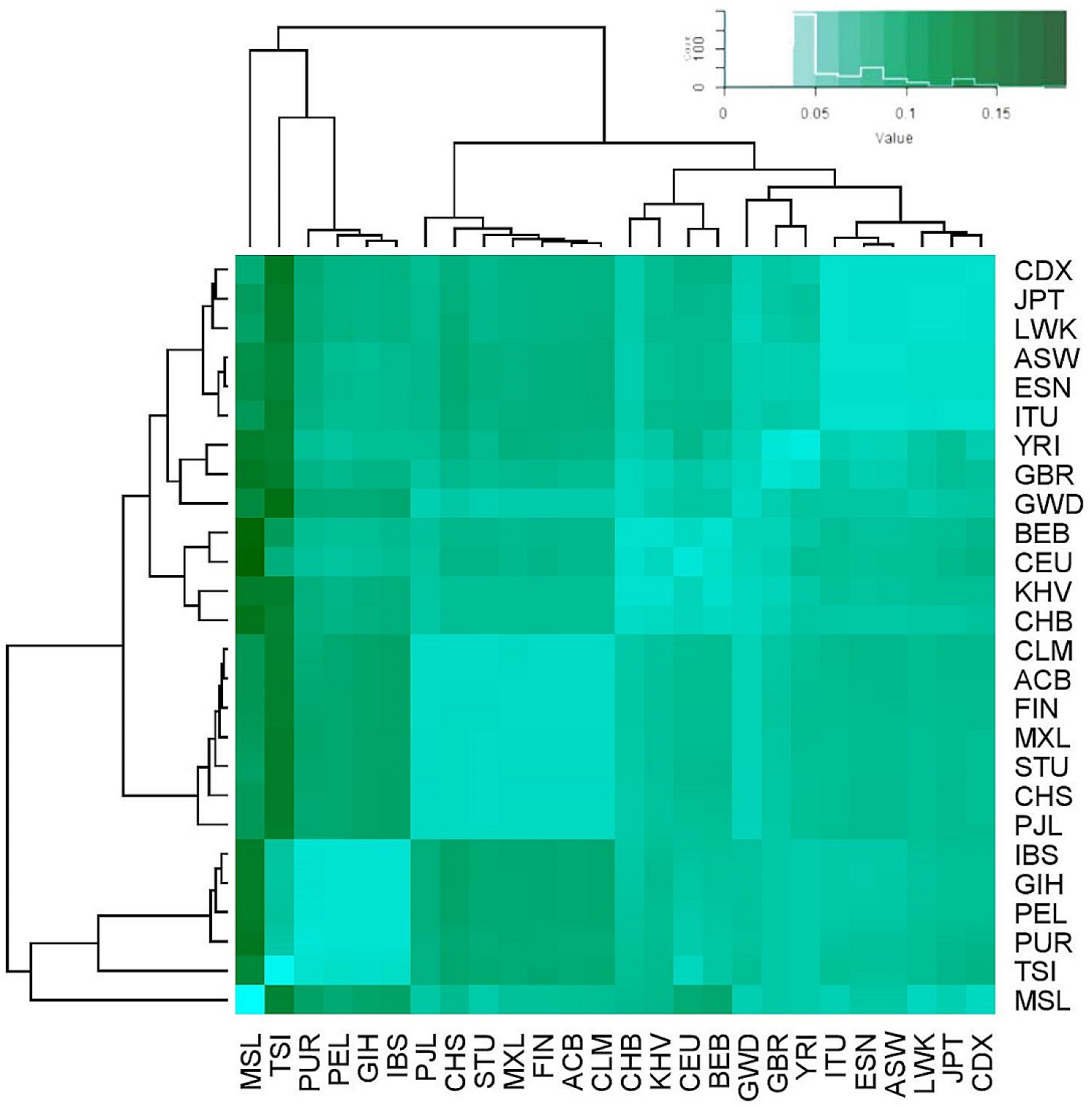
The heatmap with dendrogram representing the relationship between variants associated with DC among sub-population groups

**Fig. 4:**
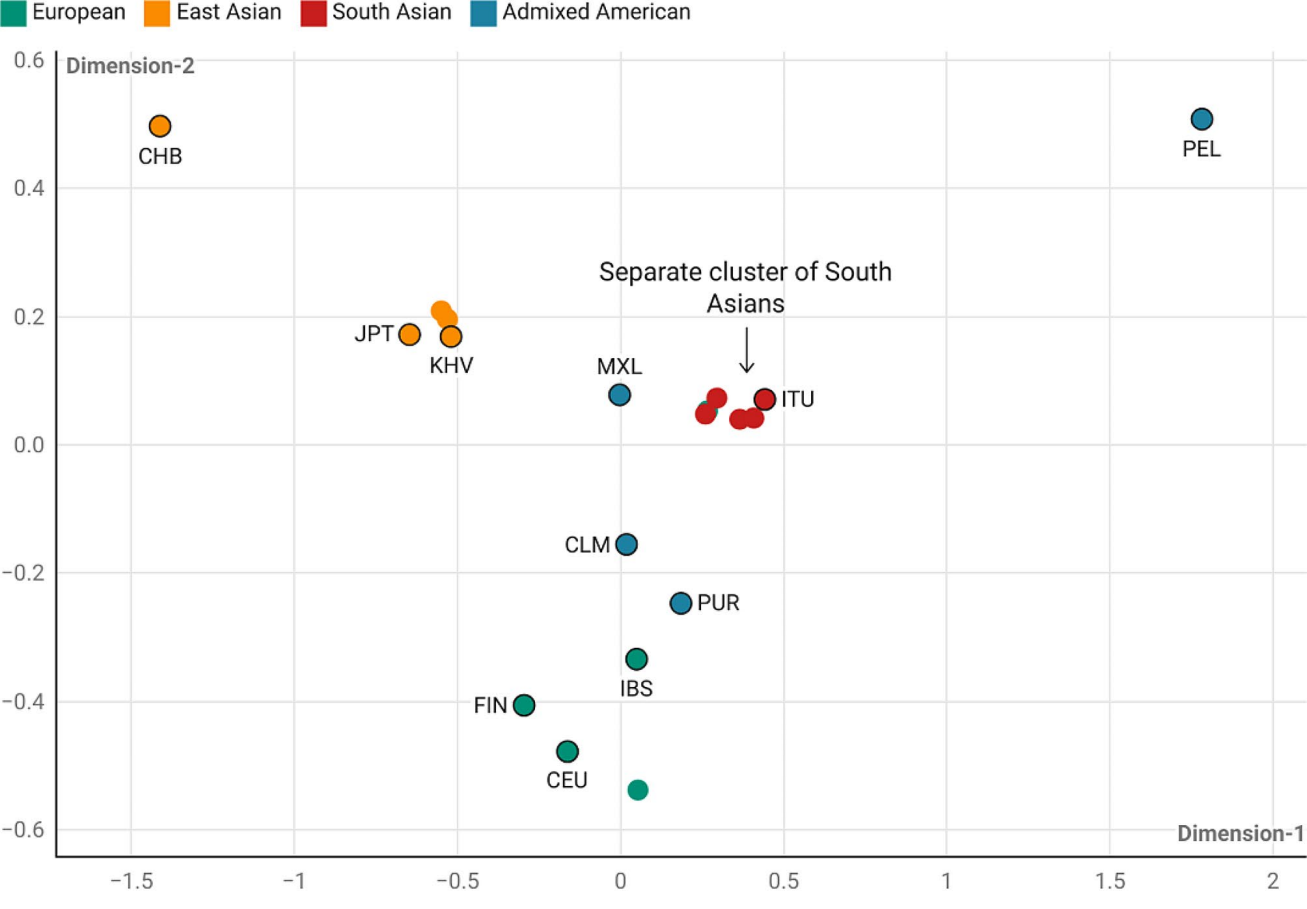
Scattered Plot depicting the effect allele frequencies of the variants identified through genome-wide association studies on stratified population groups derived from the 1000 genome data

To quantify the effect allele frequency of selected variants, the Fst were calculated for different populations. Moreover, the allele frequency was also found to differ among the SAS population groups, highlighting the importance of screening these variants in the Indian populations in association with DC. The results obtained were further plotted as a heat map and scattered plot (Figs. 3 and 4).

The heat map conjugated with the phylogenetic tree shows three clusters on the basis of F*st* values **(**Fig. 3**)**.,Interestingly, the EUR population is distinct from the other populations, as depicted by the green solid dots in the plot (Fig. 4**)**. The EAS and SAS populations were also relatively distinct and were described as orange and red solid dotes, respectively (Fig. 4**)**. The AA population in the plot is depicted in the blue solid dots and is present near the EAS, SAS, and EUR clusters, suggesting it has a genetic mix of these populations (Fig. 4**)**. The results also revealed a distinct cluster of South Asians. This observation underscores the idea that a one-size-fits-all approach for gene associations may not be suitable across all global populations. It highlights the importance of conducting ethnicity-specific GWAS or population-based case control association studies. This underscores the need to validate these genetic variations, as suggested by many other studies on different disease traits in Indians [30–32].

## Discussion

DC is the most common chronic disease caused by interaction of bacteria, genetics and environmental factors and was observed to affect both children and adults. The severeity of DC increases if not treated timely could cause disease associated with DC and may affect the quality of life. The present clinical interventions came into existence once an individual gets affected with DC, but through the discovery of various candidate genes, one can manage DC proactively by identifying individuals at higher risk and implementing preventative measures, potentially reducing the severity of the disease and improving overall quality of life. The majority of genetic studies on dental caries have evaluated genetic variations in particular genes based on their presumed or known functions that are thought to be relevant to the trait, using the standard population-based case-control approach of testing for association between specific variants or alleles at a genetic locus. The identified loci or variations could not be replicated successfully in other ethnic groups. This lack of replication is thought to be a result of genetic heterogeneity. To unravel the cause of this genetic heterogeneity, the current study employed an empirical approach to investigate the genetic variations observed within South Asian populations and their implications with respect to DC. The variations were selected based on the statistical stringency by applying the mean effect allele frequencies in SAS, EUR, EAS, and A.A.

The application of significance level of 5 × 10^− 6^ is strategically implemented to enhance the downstream analysis by including a greater number of variations to increase the statistical power. To ensures the robusrt foundation for the subsequent analysis and strengthens the overall effectiveness of the study. Additionally, variants with a minor allele frequency greater than 1% and a p-value threshold of less than 5 × 10^− 6^ were screened (Fig. 1). The selected variations were also evaluated by comparing minor allele frequencies across diverse databases (Supplementary Table 6). The consistent pattern in allele frequencies supports the effectiveness of applied selection criteria.

Moreover, comparison of variations below the GWAS threshold p-value 5 × 10^− 8^ (four variation remained) was determined and Fst was calculated. Fst results obtained aligned with our prior results (Supplementary Fig. 2), highlighting the robustness of the selection criteria.

To understand this genetic heterogeneity, we calculated the Fst [33] to measure the population differences between different global populations using the selected variations and to have insights into the evolutionary processes that influence the structure of genetic variation within and among studied populations. The results were in coherence with the previous studies on different phenotypes [30, 31]. The cluster formed by South Asian populations is entirely different than European, East Asians and Admixed Americans, signifying the difference in South Asian population groups could be an outcome of ancient admixture, or differences in effect allele frequencies among the studied population could be an outcome of a founder effect, or distribution of variants is the combined effect of both admixture and founder effects [34]. We performed functional annotation of variants and identified rs9311745 as having a relatively high CADD_PHRED score of 7.91 (Supplementary Table 3).

The results of the present study emphasise a dire need to conduct the genome-wide association of DC in Indians or South Asians. Additionally, it is pertinent to validate the established variations of DC in South Asians and other population groups. Apart from SAS, the present study observed the genetic distinction between PEL than other AA population groups and CHB than other EAS. The results from the present study highlight that the genetics of DC goes beyond simplistic categories like “Western” or “Eastern” populations. It highlights the need for detailed analysis of allele frequencies and DC genomic patterns in specific groups, such as SAS, AA, and EAS indigenous populations, instead of relying on broad and inaccurate racial stereotypes. While the present study provides insights into the genetic landscape of DC as one trait, we recognise certain areas that warrant future research and acknowledge inherent limitations. Future investigations could benefit from more targeted and refined sub-phenotyping to better delineate genetic associations specific to primary and permanent dentition and its age-specific influences on DC.

Further, clinicians DC can benefit significantly from an awareness of genetic contributions to DC as a foundation for host susceptibility. They may be able to explain to patients that certain types of caries are more strongly connected with genetic risk, explaining to the patient and the dental practitioner why identical behavioural risks, like individuals with the same eating habits, etc., have varying caries risk.

### Electronic supplementary material

Below is the link to the electronic supplementary material.

Supplementary Material 1

Supplementary Material 2

## Data Availability

All data analysed in this study were obtained from public databases as cited; no direct human data was collected.
