# Supplementary material for: Quantifying variations associated with dental caries reveals disparity in effect allele frequencies across diverse populations
Source: BMC Genom Data. 2024 Jun 3;25:50. doi: 10.1186/s12863-024-01215-z (PMC11149341; doi:10.1186/s12863-024-01215-z)
Supplement: Supplementary file 1 — Supplementary Material 1 [file 12863_2024_1215_MOESM1_ESM.docx]

**
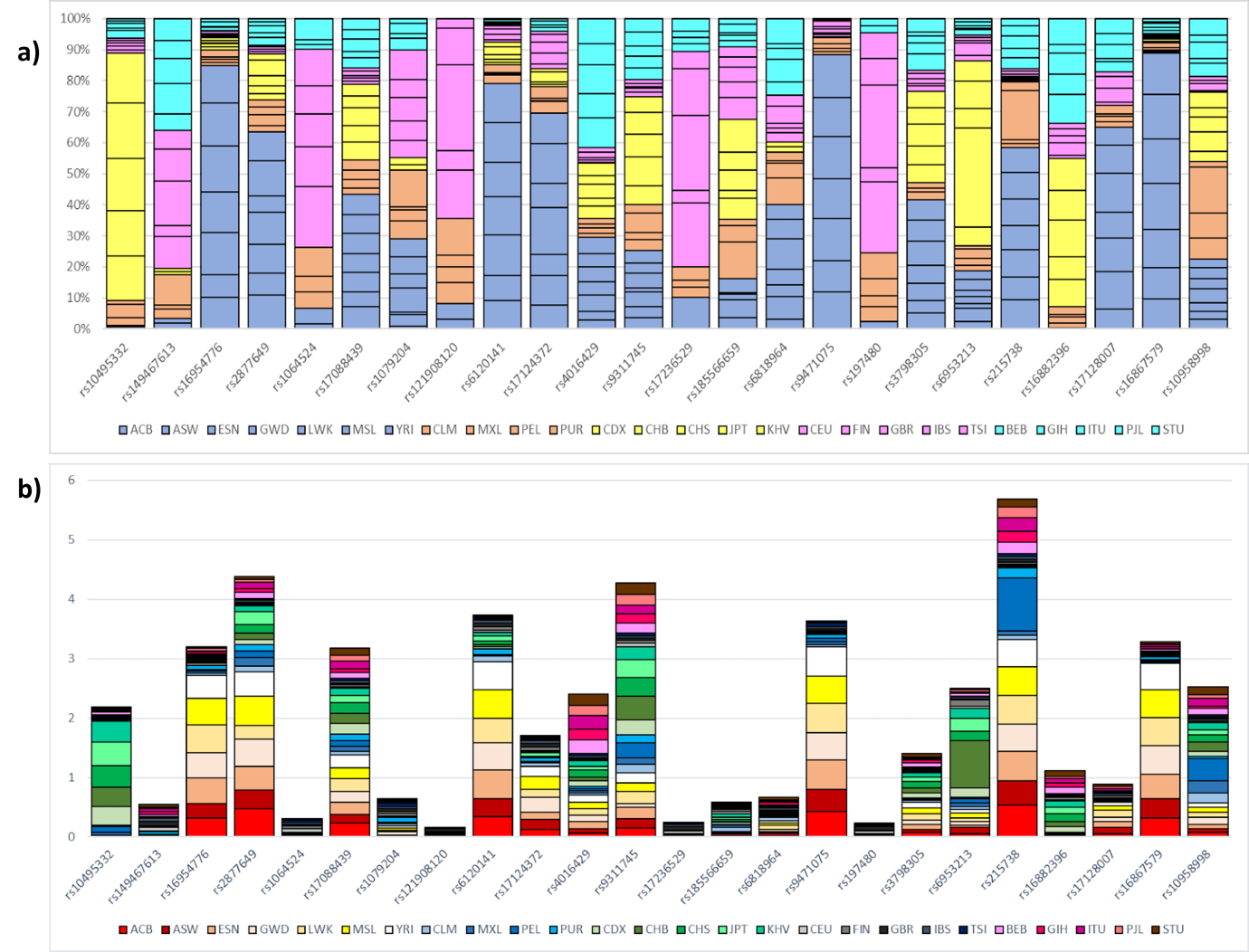
**

**Supplementary Figure S1: Effect allele frequencies observed among different population groups**

**
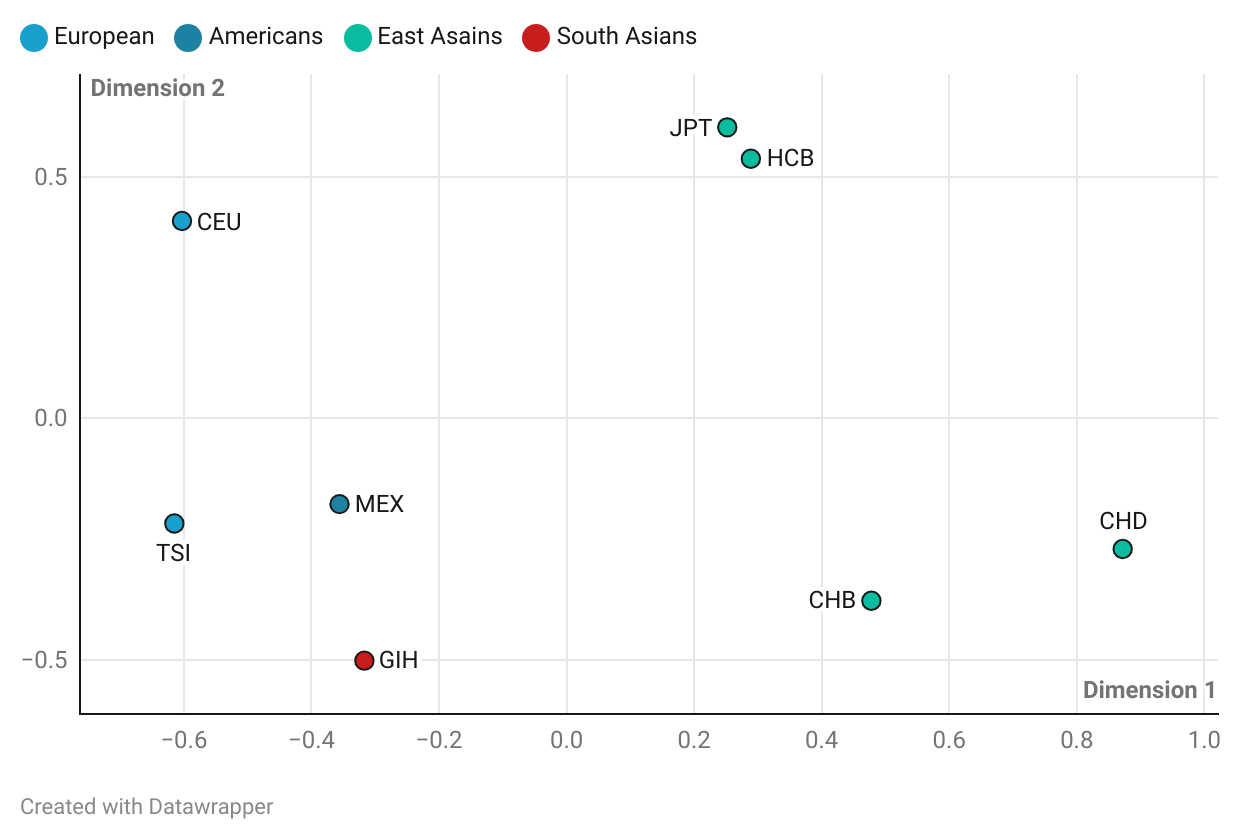
**

**Supplementary Figure S2: Scattered Plot depicting the effect allele frequencies of the variants with GWAS (P < 5x10-8) threshold on different population groups with four variants (rs17236529, rs149467613, rs121908120, rs185566659). A normalized stress value of 0.008 obtained from Multidimensional Scaling analysis is considered very low, and it indicates a highly satisfactory fit between the observed pairwise distances and the distances in the reduced-dimensional space. The observed distances in the reduced space align very well with the actual genetic distances, indicating a robust representation of population structure.**
